# Construction of a Synthetic Aniline-Degrading Consortium Consisting of *Pseudomonas* sp. RF and *Acidovorax* sp. PH Guided by Soil Niche Information from Contaminated Sites

**DOI:** 10.3390/microorganisms14030678

**Published:** 2026-03-17

**Authors:** Hui Pan, Jun Pan, Yanru Yang, Huafeng Zhong

**Affiliations:** 1 School of Municipal and Environmental Engineering, Shenyang Jianzhu University, Shenyang 110168, China; hui18309896269@163.com (H.P.);; 2 College of Engineering, Shenyang Agricultural University, Shenyang 110866, China; yangyanru@syau.edu.cn

**Keywords:** aniline, biodegradation, synthetic community, soil niche, strain screening

## Abstract

The development of effective remediation strategies for aniline-contaminated sites has become a significant research focus in environmental science. This study aimed to construct a highly efficient aniline-degrading synthetic microbial consortium guided by ecological niche information from contaminated soil. Microbial community analysis of aniline-contaminated soil from a typical industrial park revealed the significant enrichment and adaptability of Proteobacteria and its genus *Pseudomonas* in the polluted environment. Based on these ecological niche characteristics, a targeted screening strategy was employed to isolate two highly efficient degrading strains from heavily contaminated soil: *Pseudomonas* sp. RF and *Acidovorax* sp. PH. Both strains exhibited excellent aniline degradation performance in monoculture, with strain RF capable of completely degrading 1000 mg·L^−1^ aniline within 24 h. Through orthogonal experiments to optimize the inoculation ratio, a synthetic consortium, RF-PH, composed of the two strains at a 3:1 ratio, was constructed. This consortium demonstrated significant synergistic effects, with degradation efficiency markedly surpassing that of the individual strains. Specifically, its degradation rate for 500 mg·L^−1^ aniline within 12 h was 11.33–17.02% higher than that of the individual strains. This study confirms the effectiveness of a targeted screening and synthetic consortium construction strategy based on ecological niche information, providing efficient microbial resources and technical support for the bioremediation of aniline-contaminated sites.

## 1. Introduction

Aniline (C_6_H_7_N), an important aromatic compound widely used in the synthesis of rubber, pesticides, dyes, and pharmaceuticals [1], poses significant environmental concerns due to its persistence. Its resistance to natural attenuation leads to long-term retention in soils and water bodies near industrial areas [2]. For instance, concentrations as high as 50.35 mg·kg^−1^ and 132.11 mg·kg^−1^ have been detected at 10 cm and 30 cm depths, respectively, in soil from an industrial site in Perm, Russia [3]. A spill at a BASF plant in the United States resulted in soil aniline residues reaching 944 mg·kg^−1^, with groundwater concentrations up to 270 mg·L^−1^ [4]. In China, a leakage incident in Shanxi caused aniline levels in a downstream river to reach 72 mg·L^−1^ [5]. Residual aniline in the environment threatens ecosystems by exerting high toxicity on aquatic organisms, inhibiting the growth of crops such as wheat and rice, and inducing genotoxic damage in root tip cells [4]. Moreover, aniline can enter the human body via inhalation, ingestion, or dermal contact, potentially causing methemoglobinemia, hemolytic anemia, and kidney damage [6]. Due to its significant environmental and health risks, aniline has been listed as a priority pollutant by the United States and is strictly regulated in many countries and regions, including China, Japan, and the European Union. Consequently, developing efficient remediation strategies for aniline-contaminated sites has become a critical research focus in environmental science.

Among various treatment technologies, bioremediation—particularly microbial remediation—has gained attention for its cost-effectiveness, operational simplicity, and minimal secondary pollution [7]. Key microbial enzymes, such as aniline dioxygenase, initiate degradation by attacking and cleaving the stable benzene ring, ultimately converting aniline into harmless or less toxic products [8]. Several aniline-degrading microorganisms have been reported, including *Dietzia natronolimnaea* [9], *Erwinia* sp. [2], *Pigmentiphaga daeguensis* [3], and *Rhodococcus* sp. [10]. However, their practical application is often limited to low aniline concentrations, with reduced efficiency and extended adaptation periods in high-concentration scenarios. For example, *Dietzia natronolimnaea* JQ-AN, reported by Jin et al. [9], achieved only a 72% degradation of 500 mg·L^−1^ aniline after 7 days. Zhang et al. [11] observed degradation rates of 82% and 60% within 5 days for aniline concentrations of 600 mg·L^−1^ and 1000 mg·L^−1^, respectively, using *Tsuruhatensis* sp. Chen et al. [12] developed an enriched consortium dominated by *Comamonas* and *Zoogloea*, which removed only 44.1% and 61.2% of aniline on the first and second days, respectively, in an aerobic bioreactor. These findings underscore the need for more efficient aniline-degrading microbial resources.

Niche-based targeted screening offers a promising strategy for obtaining highly efficient functional strains. The underlying principle is that indigenous microbial communities in long-term polluted environments undergo adaptive succession and niche differentiation. Microbial taxa capable of utilizing the pollutant as their primary carbon, nitrogen, or energy source gain a competitive advantage and become enriched within the community [13]. Thus, areas frequently contaminated with aniline—such as those near rubber, chemical, dye, and pharmaceutical industries—are promising sources for isolating microorganisms with high resistance or degradation capacity. For instance, Liu et al. [14] isolated *Ochrobactrum* sp. MC-01 from industrial sludge, which survived in aniline wastewater at concentrations up to 6500 mg·L^−1^. Fu et al. [15] successfully isolated the aniline-degrading bacterium *Pseudomonas veronii* T1 from contaminated soil. Nevertheless, the complexity of real contaminated sites often challenges the adaptability of single strains to fluctuating environmental conditions [16]. The construction of synthetic microbial consortia by combining functionally complementary strains has emerged as a promising strategy to develop efficient biodegradation products [17]. For instance, a synthetic consortium designated ECT, composed of *Ralstonia* sp. CP, *Rhizobium* sp. BX, and *Acinetobacter* sp. FL, was shown to significantly enhance the degradation efficiency and stability of petroleum hydrocarbons in contaminated soil [18]. Similarly, a bacterial consortium constructed from *Cellulosimicrobium* sp. RS and *Brucella* sp. BZ demonstrated highly efficient degradation of polycyclic aromatic hydrocarbons [19]. These examples underscore the potential of rationally designed consortia to overcome the limitations of single strains in complex environments.

This study systematically investigated the microbial niche characteristics of aniline-contaminated soil from a typical industrial park and performed targeted screening, leading to the isolation of two highly efficient aniline-degrading strains. These strains were then rationally assembled into a stable and efficient synthetic microbial consortium for aniline degradation. The research aims to validate the feasibility of a niche-based strategy for constructing functional microbial consortia and to provide efficient microbial resources and data to support the development of bioremediation technologies for aniline pollution.

## 2. Materials and Methods

### 2.1. Materials

Aniline was purchased from Da Mao Chemical Reagent Factory (Tianjin, China). R2A medium was used with this composition: yeast extract 0.50 g·L^−1^, Proteose Peptone 0.50 g·L^−1^, Casamino acids 0.50 g·L^−1^, glucose 0.50 g·L^−1^, starch 0.50 g·L^−1^, Na-pyruvate 0.30 g·L^−1^, K_2_HPO_4_ 0.30 g·L^−1^, MgSO_4_·7H_2_O 0.05 g·L^−1^. MSM medium was used with this composition: MgSO_4_·7H_2_O (0.2 g·L^−1^), CaCl_2_ (0.01 g·L^−1^), FeSO_4_·7H_2_O (0.001 g·L^−1^), KH_2_PO_4_ (1.5 g·L^−1^), Na_2_HPO_4_ (1.00 g·L^−1^), MnSO_4_ (0.02 g·L^−1^). And NH_4_Cl (1 g·L^−1^). LB medium was used with this composition: tryptone 10 g·L^−1^, yeast extract 5 g·L^−1^, NaCl 10 g·L^−1^. The reagents used above are also from Da Mao Chemical Reagent Factory (Tianjin, China).

### 2.2. Soil Samples

Experimental soil samples were collected from a chemical industrial park in Dalian City, Liaoning Province, China (39.40° N, 121.75° E). Based on the degree of aniline contamination, the soils were categorized into: heavily polluted soil (HS) (aniline concentration 1381.17 ± 34.26 mg·kg^−1^), lightly polluted soil (LS) (aniline concentration 316.49 ± 17.28 mg·kg^−1^), and clean soil (CS) (no aniline detected). For each soil type, three independent sampling points were collected, resulting in a total of nine soil samples. The basic physical and chemical properties of soils in each group are shown in Table S1.

### 2.3. DNA Extraction and Microbial Community Analysis

Genomic DNA was extracted from each sample following the method described by Jing et al. [18]. Subsequently, the V3 + V4 region of the bacterial 16S rDNA was amplified using barcoded specific primers. The primer sequences were: 341F: CCTACGGGNGGCWGCAG; 806R: GGACTACHVGGGTATCTAAT. The purified amplicons were ligated with sequencing adapters to construct sequencing libraries, which were then sequenced on an Illumina platform. The sequencing service was performed by Shanghai Meiji Company (Shanghai, China). Raw sequencing data were processed using the UPARSE pipeline to remove chimeras, and sequences were clustered into operational taxonomic units (OTUs) at a 97% similarity threshold. Further statistical analyses were performed using this rarefied OTU table. The raw sequences have been deposited in the NCBI Sequence Read Archive (SRA) under accession number PRJNA1186943. Data analysis was conducted on the free online Majorbio Cloud Platform (www.majorbio.com). Microbial co-occurrence networks were constructed based on Spearman’s rank correlation coefficients (r) calculated from the relative abundances of genera. Only correlations with r > 0.6 and statistical significance (*p* < 0.05) were retained to build the network. The networks were visualized using Gephi software (version 0.10.1).

### 2.4. Strain Isolation and Identification

Aniline-degrading bacteria were isolated from soil samples contaminated with aniline in a high-concentration area. Specifically, based on the community analysis results, after identifying the target functional bacterial genus, the targeted screening medium R2A (pH 7.0, composition: yeast extract 0.50 g·L^−1^, Proteose Peptone 0.50 g·L^−1^, Casamino acids 0.50 g·L^−1^, glucose 0.50 g·L^−1^, starch 0.50 g·L^−1^, Na-pyruvate 0.30 g·L^−1^, K_2_HPO_4_ 0.30 g·L^−1^, MgSO_4_·7H_2_O 0.05 g·L^−1^) was determined using KOMODO (the Known Media Database, https://mediadive.dsmz.de/ (accessed on 10 September 2025)). The soil sample was serially diluted and plated onto R2A agar plates supplemented with 1000 mg·L^−1^ aniline for bacterial cultivation. After 3 days of incubation, fully grown colonies were picked and cultured in R2A broth containing 1000 mg·L^−1^ aniline. Following nearly one month of screening and enrichment, two colonies were isolated, cultured, and maintained as pure cultures, designated RF and PH. Universal primers 27F and 1492R were used to amplify the 16S rRNA genes of the strains, and the amplicons were sequenced by Sangon Biotech Co., Ltd. (Shanghai, China). The 16S rRNA gene sequences of strains PH and RF have been deposited in GenBank under accession numbers PX736765 and PX736766. Multiple sequence alignment of the 16S rRNA gene sequences was performed using Clustal X (version 1.83). The phylogenetic tree was then constructed using the neighbor-joining method in MEGA software (version 6.06) with 1000 bootstrap replicates to assess branch support.

### 2.5. Determination of Aniline Degradation Function by Strains

To investigate the degradation of aniline by individual strains, seed cultures (5%, *v*/*v*) were inoculated into a mineral salts medium [19] (pH 7.0) containing 500 mg·L^−1^ aniline as the sole carbon source, with the following components: MgSO_4_·7H_2_O (0.2 g·L^−1^), CaCl_2_ (0.01 g·L^−1^), FeSO_4_·7H_2_O (0.001 g·L^−1^), KH_2_PO_4_ (1.5 g·L^−1^), Na_2_HPO_4_ (1.00 g·L^−1^), MnSO_4_ (0.02 g·L^−1^), and NH_4_Cl (1 g·L^−1^). The cultures were incubated under continuous shaking at 30 °C for 72 h. Batch experiments were performed to examine the effects of substrate concentration (100–1000 mg·L^−1^) and pH (5.0–9.0) on aniline degradation by the strains. All experiments were conducted in triplicate, and mean values were calculated.

### 2.6. Microbial Consortium Construction

Referring to the synthetic microbial community method described by Jing et al. [20], the optimal combination ratio of strains PH and RF was investigated. Specifically, strains were cultivated in LB medium to the late logarithmic growth phase, harvested by centrifugation, and washed with sterile water. The cell pellets were then resuspended in sterile water and adjusted to an optical density (OD_600_) of 2.0 (approximately equivalent to 1.0 × 10^9^ CFU·mL^−1^ for both strains, as determined by preliminary plate counting). The optimal inoculation ratio between the two strains was determined through an orthogonal experimental design. In 100 mL of mineral salt medium containing 500 mg·L^−1^ aniline, bacterial resuspensions were added according to the ratios outlined, followed by shake-flask cultivation at 150 r·min^−1^ for 3 days. Based on the aniline degradation rate, the optimal combination ratio was selected to construct the mixed microbial consortium. The formula for calculating aniline degradation rate (r/%) is as follows:

(1) $$r\left(\%\right)=\left(1-\frac{C_{residual}}{C_{initial}}\right)\times 100\%$$

where C*_initial_* is the initial aniline concentration (500 mg·L^−1^) and C*_residual_* is the concentration after 3 days of degradation.

### 2.7. Analytical Methods

Bacterial growth was determined by measuring protein concentration using the Bradford method [20]. This method can avoid the potential interference of colored metabolic intermediates, and it is a common method to monitor cell growth in the process of aromatic compound degradation [19]. Aniline concentration was quantified by Gas Chromatography (GC 6890, Agilent, Santa Clara, CA, USA) [21]. Specifically, the flame ionization detector (FID) was used. Separation was performed on an HP-5MS UI capillary column (30 m × 0.25 mm i.d. × 0.25 μm film thickness). High-purity nitrogen was used as the carrier gas at a constant flow rate of 1.0 mL·min^−1^. The FID was operated with hydrogen at 30 mL·min^−1^ and air at 250 mL·min^−1^. Injection was performed in splitless mode with an injection volume of 1.0 μL, and the injector temperature was maintained at 290 °C. The oven temperature program was set as follows: initial temperature 40 °C, then increased to 300 °C at a rate of 10 °C·min^−1^ and held for 5 min. Soil pH, soil organic carbon (SOC), total nitrogen (TN), and total phosphorus (TP) were determined using standard methods [22]. The morphology of the strains was observed by Scanning Electron Microscopy. In particular, bacterial cells were collected by centrifugation (10,000 r·min^−1^, 4 °C, 15 min), fixed with 5% glutaraldehyde at 4 °C for 2 h, and then dehydrated through a graded ethanol series (30%, 50%, 70%, 90%, and 100%, *v*/*v*). After freeze-drying and gold sputter-coating, the samples were observed using a scanning electron microscope (FlexSEM1000, Hitachi, Tokyo, Japan).

### 2.8. Statistical Analysis

All experiments were performed in triplicate (biological replicates), and data were expressed as the mean ± standard deviation (SD). Statistical analyses were conducted using SPSS software (version 26.0, IBM, New York, NY, USA). Comparisons among multiple groups were performed using one-way analysis of variance (ANOVA) followed by Tukey’s honest significant difference (HSD) post hoc test and *p* < 0.05 was considered statistically significant.

## 3. Results and Discussion

### 3.1. Characteristics of Microbial Community Structure in Aniline-Contaminated Site

**Figure 1:**
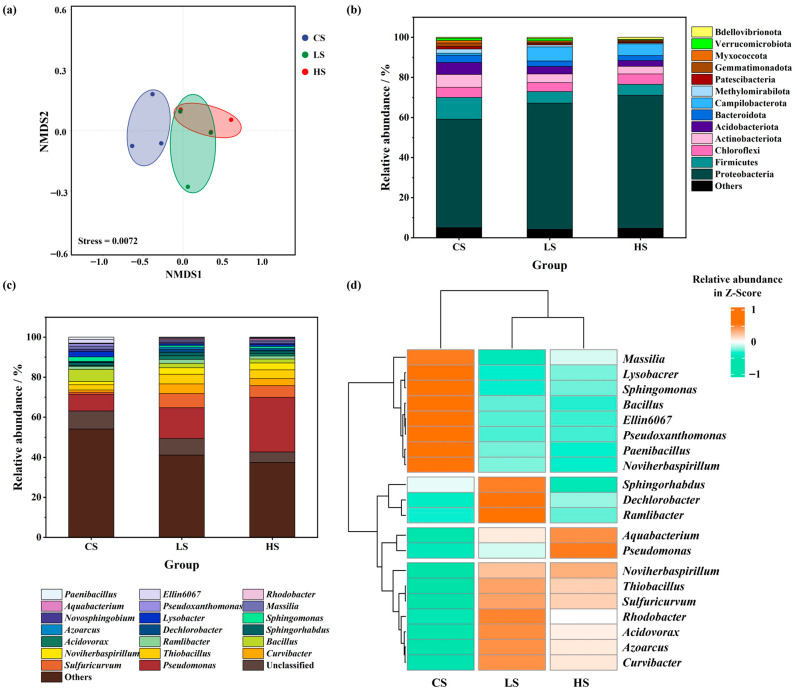
(**a**) Non-metric multidimensional scaling (NMDS) analysis of soil community structure; (**b**) soil community structure at the phylum level; (**c**) soil community structure at the genus level; (**d**) heatmap of dominant species at the genus level.

Soils from the contaminated site were divided into three zones according to the level of pollution: heavily polluted soil (HS), lightly polluted soil (LS), and clean soil (CS). The soil communities in each region were analyzed by 16S rRNA gene amplicon high-throughput sequencing. Alpha diversity was used to characterize the species diversity within microbial communities of each sample [23]. As shown in Table 1, this study calculated various alpha diversity indices, including Chao1, Shannon, Simpson, and Coverage. The alpha diversity indices indicated that the soil microbial diversity was highest in the CS group, while it decreased in the LS and HS groups. Notably, statistical analysis revealed no significant difference in alpha diversity indices between the LS and HS groups (*p* < 0.05). This suggests that aniline contamination reduces the diversity of soil microbial communities; however, at this site, the reduction effect on community diversity did not differ markedly between low and high pollution levels. Non-metric multidimensional scaling (NMDS) analysis of the communities also yielded consistent results (Figure 1a). The distances between points reflect the degree of differences in community structure, with shorter distances indicating higher community similarity [19]. The community structures of soil microorganisms in LS and HS were similar. These results imply that microorganisms in soils with different levels of aniline contamination may be capable of adapting to soils with other contamination levels, though this requires further verification by comparing the dominant species in each group.

The results of bacterial community composition analysis at the phylum level are shown in Figure 1b, where phyla with a relative abundance below 1% were grouped into the “Others” category. The abundance of Proteobacteria was the highest across all three soil regions (CS, LS, and HS), accounting for 54.06%, 63.00%, and 66.42%, respectively. Moreover, its abundance exhibited a trend of HS > LS > CS, indicating that bacteria within this phylum are more adaptable to aniline contamination. Huang et al. [24] reported that the relative abundance of Proteobacteria in aniline-contaminated soil reached 25.54%, while He et al. [6] found it to be as high as 37.69% in an aniline-polluted environment. These findings are consistent with the results of this study, suggesting that Proteobacteria exhibits strong tolerance to aniline. Therefore, bacteria from this phylum should be prioritized in the screening of highly efficient aniline-degrading microorganisms.

At the genus level, the analysis of bacterial community composition is presented in Figure 1c, with genera having a relative abundance below 1% merged into the “Others” category. *Pseudomonas* was the most abundant genus in all three soil regions (CS, LS, and HS), with proportions of 8.38%, 15.32%, and 27.24%, respectively. Its abundance displayed a trend of HS > LS > CS, indicating that this genus possesses a higher adaptability to aniline contamination. Wu et al. [25] found that *Pseudomonas* exhibits strong adaptability to aniline stress. Jiang et al. [10] reported that *Pseudomonas* serves as a core degrader in aniline degradation systems and is not easily replaced during succession. Notably, *Pseudomonas* belongs to the phylum Proteobacteria, aligning with the earlier analysis. Hence, bacteria from this genus should be considered a primary target for screening highly efficient aniline-degrading strains.

Cluster analysis (with row normalization) was performed on the dominant genera in each group, and the significance of differences between groups was evaluated (Figure 1d). The selected genera generally reflect the distribution characteristics of the microbial communities in each group. Genera such as *Sphingomonas*, *Lysobacter*, *Novosphingobium*, *Massilia*, *Pseudoxanthomonas*, *Rhodobacter*, *Ellin6067*, and *Paenibacillus* showed higher abundance in the CS group compared to the LS and HS groups, suggesting that these genera were succeeded in the community under the stress of aniline contamination. In contrast, genera including *Sulfuricurvum*, *Curvibacter*, *Thiobacillus*, *Bacillus*, *Ramlibacter*, *Acidovorax*, *Sphingorhabdus*, *Dechlorobacter*, and *Azoarcus* exhibited an abundance trend of LS > HS > CS, indicating potential aniline tolerance. For instance, *Sulfuricurvum* has been reported to have the potential to degrade various cyclic organic compounds such as polycyclic aromatic hydrocarbons and polychlorinated biphenyls [26]. Li et al. [27] observed significant enrichment of *Curvibacter* in low-concentration aniline-contaminated environments, where it was active during the initial stages of aniline degradation. Lin et al. [28] found that Thiobacillus evolved from a non-dominant to a dominant genus under aniline contamination stress. Cui et al. [29] noted that while *Bacillus* could survive long-term in aniline-contaminated environments, its abundance decreased with increasing aniline concentration. *Ramlibacter* has been reported to occupy a dominant position in aniline-contaminated environments [30]. Li et al. [31] demonstrated that *Acidovorax*, possessing denitrification capabilities, can stably exist in aniline-polluted settings. Chen et al. [32] reported that *Sphingobacterium* could adapt to aniline-contaminated environments, although its abundance declined when aniline concentrations increased rapidly. Li et al. [27] found that *Dechloromonas* was enriched in low-concentration aniline-contaminated environments, indicating tolerance to low levels of aniline. Zhang et al. [33] highlighted the important role of *Azoarcus* in promoting degradation within aniline degradation reaction systems. In addition to *Pseudomonas*, genera such as *Aquabacterium* and *Noviherbaspirillum* also exhibited an abundance trend of HS > LS > CS, suggesting that, like *Pseudomonas*, these genera may possess strong tolerance to high concentrations of aniline. Xiao et al. [34] reported the long-term stable presence of *Noviherbaspirillum* and *Aquabacterium* in aniline-contaminated environments, which aligns with the findings of this study. In summary, the dominant genera identified in the LS and HS groups likely possess aniline-degrading capabilities.

### 3.2. Inter-Species Relationships Within the Indigenous Community and Correlations Between Dominant Species and Environmental Factors

**Figure 2:**
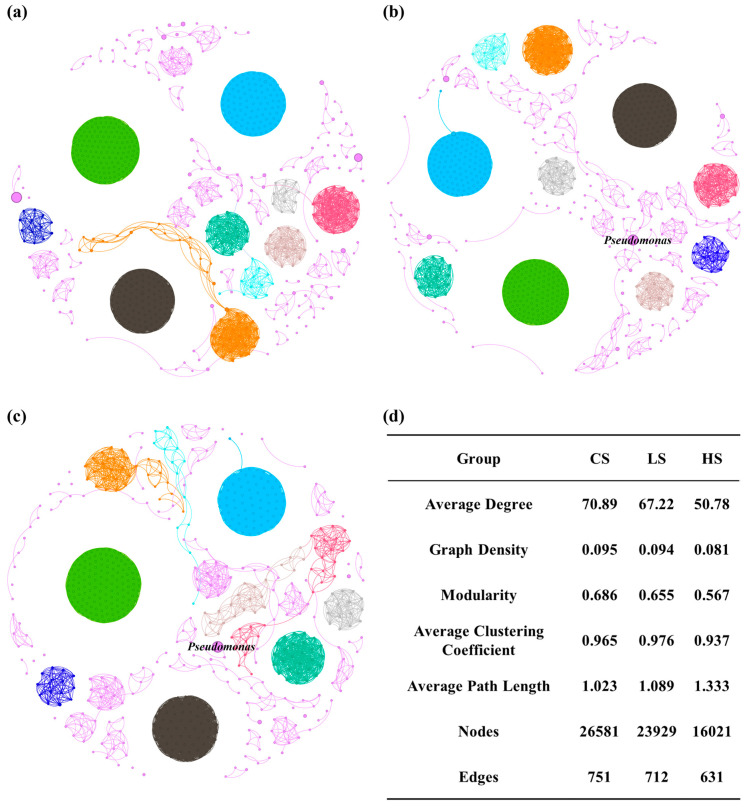
Soil microbial community co-occurrence networks. (**a**) Clean soil (CS) group; (**b**) lightly polluted soil (LS) group; (**c**) heavily polluted soil (HS) group; (**d**) topological parameters. Nodes represent different bacterial genera and are colored according to modularity classes, indicating different modules within each network.

Network analysis serves as an effective tool for revealing potential mechanisms of interaction among environmental microorganisms [35]. In this study, microbial co-occurrence networks were constructed for soils from each contaminated zone (Figure 2). As shown in the co-occurrence networks (Figure 2a–c), the microbial communities in the three soil groups exhibited distinct modular structures. In the LS and HS networks, *Pseudomonas* occupied a central position within its respective module, as indicated by its high connectivity and node size (reflecting its high relative abundance). This suggests that *Pseudomonas* may play a key ecological role in maintaining community stability under aniline stress and could be a core functional genus in the degradation process.

The average weighted degree followed the order of HS < LS < CS, indicating that aniline contamination stress reduced the complexity of the soil microbial interaction network. In microbial networks, modules are regarded as functional units, where species within the same module often occupy similar ecological niches. The modularity index was highest in the CS group (0.686), followed by the LS group (0.655), and lowest in the HS group (0.567), supporting the above inference. *Pseudomonas* tended to be more central within its respective module in the networks of both the LS and HS groups, suggesting that it may occupy a core functional role in aniline-contaminated soil systems.

**Figure 3:**
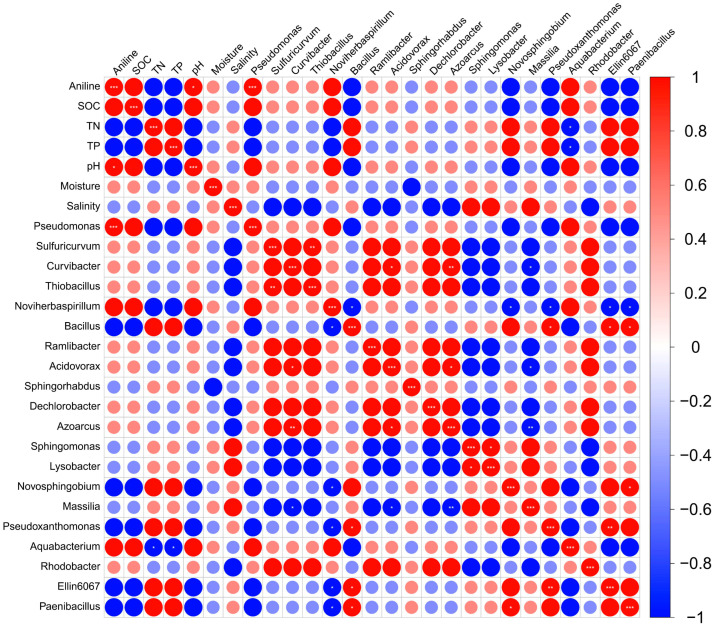
Correlation between dominant species and environmental factors. * *p* < 0.05; ** *p* < 0.01; *** *p* < 0.001.

Further analysis of the correlation between dominant soil genera and environmental factors is presented in Figure 3. The abundance of *Pseudomonas* showed a significant positive correlation with aniline concentration (*p* < 0.05), providing additional evidence that it likely possesses strong aniline tolerance and exhibits aniline-degrading potential in contaminated soils. Therefore, bacteria from this genus were selected as the primary target for subsequent screening of highly efficient aniline-degrading strains.

### 3.3. Isolation, Identification, and Functional Characterization of Highly Efficient Aniline-Degrading Bacteria

**Figure 4:**
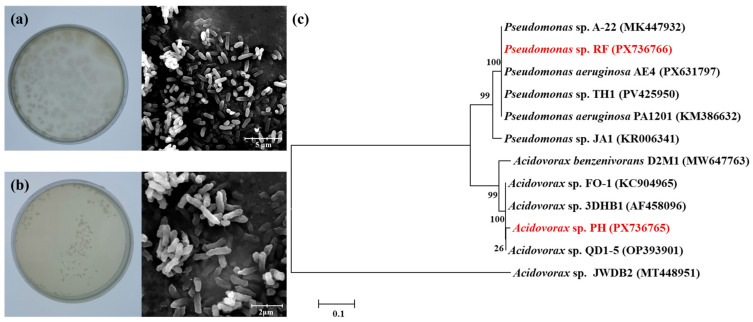
Plate and electron microscopy images of the strains ((**a**): *Pseudomonas* sp. RF; (**b**): *Acidovorax* sp. PH). (**c**) Phylogenetic tree of the strains.

Based on the aforementioned community analysis results, the genus *Pseudomonas* was identified as the primary target functional genus for screening. A targeted screening medium was determined using the KOMODO method, leading to the isolation of two strains from HS group soil capable of utilizing aniline as the sole carbon source. These strains were designated RF and PH, respectively. The colonial and cellular morphologies of the two strains are shown in Figure 4a,b, respectively. Identification via 16S rRNA gene sequencing and BLAST analysis confirmed the strains belonged to the genera *Pseudomonas* and *Acidovorax*, respectively. A phylogenetic tree was constructed for both strains (Figure 4c), revealing homology with several previously reported aniline-degrading functional strains. For instance, *Pseudomonas* sp. JA1 has been reported to completely degrade 800 mg·L^−1^ aniline within 24 h [36]. Bedics et al. [37] isolated *Acidovorax benzenivorans* D2M1, which exhibits strong aromatic compound degradation capabilities. Both strains, *Pseudomonas* sp. RF and *Acidovorax* sp. PH, therefore possess the potential to degrade aniline contaminants.

**Figure 5:**
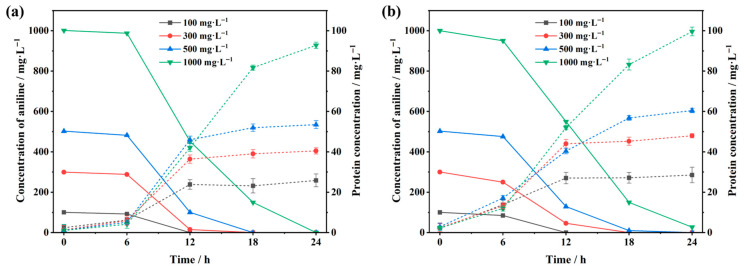
Growth and degradation curves at different substrate concentrations ((**a**): *Pseudomonas* sp. RF; (**b**): *Acidovorax* sp. PH).

Specific shake-flask experimental results demonstrated that strains RF and PH exhibited excellent degradation performance across various aniline concentrations. *Pseudomonas* sp. RF completely degraded 500 mg·L^−1^ aniline within 18 h and achieved complete degradation of 1000 mg·L^−1^ aniline within 24 h (Figure 5a). Meanwhile, *Acidovorax* sp. PH achieved 100% and 97.31% degradation rates for 500 mg·L^−1^ and 1000 mg·L^−1^ aniline, respectively, within 24 h (Figure 5b). In comparison to existing research reports, *Pseudomonas veronii* T1 required 132 h to completely degrade 1000 mg·L^−1^ aniline [15], *Delftia tsuruhatensis* AD4 showed a 93% degradation rate for 600 mg·L^−1^ aniline after 48 h [38], and *Candida tropicalis* AN1 achieved a 93.9% degradation rate for 400 mg·L^−1^ aniline within 18 h [39]. Consequently, *Pseudomonas* sp. RF and *Acidovorax* sp. PH demonstrate highly efficient aniline degradation capabilities.

### 3.4. Construction of a High-Efficiency Aniline-Degrading Microbial Consortium

To further enhance the efficiency of aniline degradation, strains *Pseudomonas* sp. RF and *Acidovorax* sp. PH were combined to form a synthetic microbial consortium. The aniline degradation capabilities of consortia with different inoculation ratios are presented in Table 2. The maximum aniline degradation rate obtained in the experiments was 93.28%, from Experiment No. 2, where the inoculation ratio of strains RF to PH was 3:1. This indicates that the aniline degradation rate reaches its maximum when the biomass inoculation ratio is RF:PH = 3:1. The consortium at this optimal ratio was designated RF-PH.

**Figure 6:**
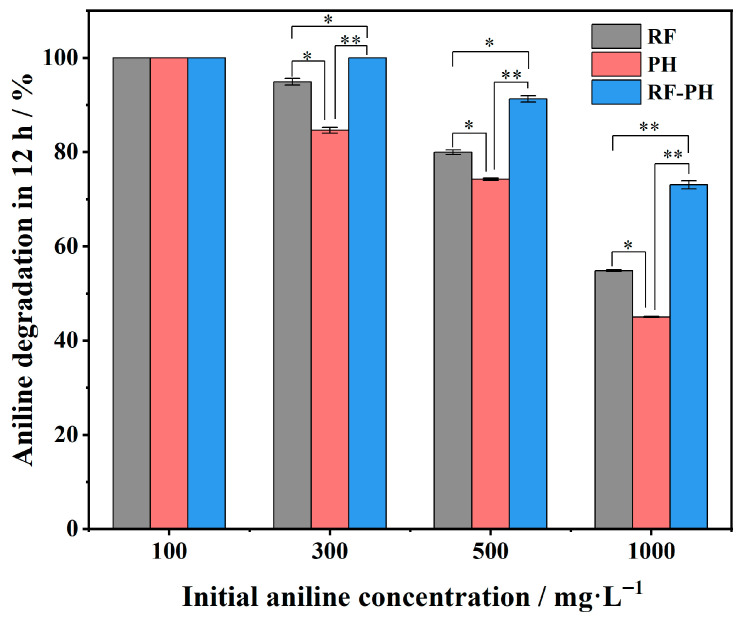
Comparison of aniline degradation rates between the synthetic consortium RF-PH and individual strains. Asterisks indicate statistically significant differences (*: *p* < 0.05, **: *p* < 0.01).

The degradation efficiency of the synthetic consortium RF-PH was compared with that of the individual strains, as shown in Figure 6. The degradation efficiency of the synthetic consortium RF-PH was significantly improved compared to that of the individual strains. In shake-flask experiments, the consortium RF-PH completely degraded 300 mg·L^−1^ aniline within 12 h. For 500 mg·L^−1^ aniline, the degradation rate reached 91.28% within 12 h, representing an increase of 11.33% and 17.02% compared to the degradation rates achieved by strain RF or strain PH alone, respectively. For 1000 mg·L^−1^ aniline, the degradation rate reached 73.06% within 12 h, representing an increase of 18.25% and 28.04% compared to the degradation by strain RF or strain PH alone, respectively. These results demonstrate that the synthetic consortium RF-PH possesses a highly efficient aniline degradation capability.

The enhanced degradation efficiency of the synthetic consortium RF-PH may be attributed to cooperative and complementary characteristics of the two strains. Both *Pseudomonas* sp. RF and *Acidovorax* sp. PH exhibited rapid adaptation to aniline stress and efficient degradation during the early growth phase. Their combination allowed for both rapid initial removal and sustained degradation, suggesting that the consortium may possess greater collective tolerance to substrate toxicity. Furthermore, as the two strains belong to different genera, they may occupy complementary ecological niches, potentially reducing competition and enabling more efficient resource utilization, and possibly even synergistic degradation effects. Further studies at the molecular level would be valuable to fully elucidate the underlying mechanisms.

## 4. Conclusions

This study successfully isolated two highly efficient aniline-degrading strains, *Pseudomonas* sp. RF and *Acidovorax* sp. PH, from an aniline-contaminated site by integrating soil microbial ecological niche analysis with targeted screening. Furthermore, a synthetic microbial consortium, RF-PH, with synergistic degradation capabilities was constructed based on these isolates. At the optimal inoculation ratio (RF:PH = 3:1), the consortium demonstrated significantly enhanced degradation efficiency for various aniline concentrations compared to the individual strains, highlighting the advantages of synthetic consortia in improving pollutant degradation rates and system stability. Mechanistic studies indicated that the consortium primarily mineralizes aniline via the catechol pathway. These results validate the feasibility of a strategy that guides functional strain screening and rational consortium construction based on in situ ecological niche information, offering a novel approach for developing targeted and highly efficient microbial remediation technologies. Future research should further investigate the colonization capacity, long-term stability, and interactions with indigenous microorganisms of this synthetic consortium in complex, real-world contaminated soils to facilitate its transition from laboratory to engineering applications.

## Figures and Tables

**Table 1:** α diversity of soil communities.

Group	CS	LS	HS
Chao	2907.93 ± 50.61 a	2226.52 ± 367.05 b	2115.45 ± 709.83 b
Shannon	6.11 ± 0.18 a	5.33 ± 0.38 b	4.94 ± 0.78 b
Simpson	0.01 ± 0.01 a	0.03 ± 0.01 b	0.06 ± 0.03 b
Coverage	0.9880 ± 0.0007 a	0.9919 ± 0.0019 b	0.9914 ± 0.0042 b

Note: Different letters indicate differences between groups (*p* < 0.05).

**Table 2:** Orthogonal test of strain ratio.

No.	Dosage of Strain (mL)		Strain Ratio	Aniline Degradation Rate (*r*/%)
	A (RF)	B (PH)		
1	1	2	1:2	89.03%
2	3	1	3:1	93.28%
3	2	2	1:1	91.47%
4	2	3	2:3	79.85%
5	1	3	1:3	91.32%
6	2	1	2:1	89.00%
7	1	1	1:1	87.17%
8	3	2	3:2	88.02%
9	3	3	1:1	84.32%

## Data Availability

The original contributions presented in this study are included in the article/Supplementary Materials. Further inquiries can be directed to the corresponding author.

## Supplementary Materials

The following supporting information can be downloaded at: https://www.mdpi.com/article/10.3390/microorganisms14030678/s1, Table S1: Physical and chemical properties of the soil.
