# Supplementary material for: Construction of a Synthetic Aniline-Degrading Consortium Consisting of Pseudomonas sp. RF and Acidovorax sp. PH Guided by Soil Niche Information from Contaminated Sites
Source: Microorganisms. 2026 Mar 17;14(3):678. doi: 10.3390/microorganisms14030678 (PMC13029111; doi:10.3390/microorganisms14030678)
Supplement: Supplementary file 1 [file microorganisms-14-00678-s001.zip › microorganisms-4182321-supplementary.pdf]

Supplementary Materials

Table S1 Physical and chemical properties of the soil

Group	CS	LS	HS
Aniline / mg·kg^{-1}	0	316.49±17.28	1381.17±34.26
SOC / g·kg^{-1}	5.88±0.14	5.94±0.11	6.71±0.20
TN / g·kg^{-1}	0.64±0.02	0.49±0.03	0.42±0.02
TP / g·kg^{-1}	0.24±0.01	0.21±0.01	0.19±0.01
pH	7.38±0.02	7.39±0.01	7.43±0.01
Moisture	12.66%±0.64%	12.43%±1.04%	13.02%±0.55%
Salinity / g·kg^{-1}	4.81±0.37	4.63±0.42	4.68±0.39
